# Current patterns of genetic diversity in indigenous and introduced species of land snails in Cameroon reflect isolation by distance, limited founder size and known evolutionary relationships

**DOI:** 10.1080/23802359.2017.1347837

**Published:** 2017-07-07

**Authors:** Ivo N. Woogeng, Willem G. Coetzer, Kingsley A. Etchu, Kenneth J.N. Ndamukong, J. Paul Grobler

**Affiliations:** a Department of Zoology and Animal Physiology, University of Buea, Buea, Cameroon;; b Department of Genetics, University of the Free State, Bloemfontein, South Africa;; c Institute of Agricultural Research for Development (IRAD), Ekona, Cameroon

**Keywords:** Population structure, isolation by distance, conservation, genetic diversity, edible land snails

## Abstract

The aim of this study was to gain molecular insights into patterns of genetic diversity in indigenous and introduced land snails in Cameroon. These species, *Archachatina marginata* and *Achatina fulica,* form an important source of protein in Cameroon, but widespread utilization may possibly impact natural patterns of genetic diversity of the indigenous species, while the introduced species may display signs of genetic drift. The evolutionary relationship between the indigenous and introduced species was also studied. Specimens were collected from seven sites in Cameroon. Genetic analyses using COI mitochondrial DNA data suggest that gene flow among the *Ar. marginata* populations screened follows a model of isolation by distance, and genetic diversity estimates for this species did not provide support for the hypothesis of loss of genetic diversity in areas of intense harvesting. Diversity in the introduced species was much lower, which is likely the signature of an introduction involving limited numbers.

African land snails of the genera *Archachatina* and *Achatina* are the most widely harvested edible snails in West African forest zones (Ajayi et al. 1978; Ogunsanmi et al. 2003). A study of genetic diversity in land snails will be informative from several perspectives. Firstly, declining numbers, population fragmentation, local extinction, translocation, and domestication may ultimately result in genetic effects such as inbreeding, drift and the disruption of natural patterns of genetic structure in indigenous populations. Secondly, land snails are good models for studying population genetic processes such as connectivity in species with limited dispersal ability (see reviews by Pfenninger and Posada 2002; Kappes and Schilthuizen 2014). Finally, *Archachatina* in Cameroon is found in close proximity to *Achatina (Lissachatina) fulica*, an east African native that is the world’s most important invasive land mollusk (Raut and Barker 2002). Genetic diversity in *Ac. fulica* has been studied and it was reported that it has restricted genetic variation in populations outside of East Africa and the Indian Ocean Islands (Fontanilla et al. 2014). Patterns of diversity in *Ac. fulica* is thus potentially guided by the same influences as that affecting *Archachatina*, but with the added complications associated with a small founder group. The current study therefore focused on the phylogeography, genetic diversity and evolutionary relationship of *Ar. marginata* (Swainson, 1821) and the highly invasive *Ac. fulica* (Férussac, 1821) within an unstable Cameroonian natural environment characterized by habitat fragmentation and disturbed habitat.

A total of 70 snails were collected from five of the six divisions of the southwestern regions of Cameroon (*Ar. marginata* = 31; *Ac. fulica* = 39; Supplemental Table S1). Only sexually mature adult snails (characterized with a lip at the edge of the shell aperture) were collected. Tissues were stored in 95% alcohol and transported to the University of the Free State, South Africa, for DNA extraction and sequencing. Import and export permits were duly obtained from the relevant authorities. Samples were stored at −20 °C, upon arrival in South Africa. DNA was then isolated using the Roche High Pure PCR Template Preparation Kit (Roche Diagnostics) and standard protocols for DNA isolation from tissue samples. For genetic analyses, a portion of the mitochondrial DNA (mtDNA) cytochrome c oxidase 1 (CO1) gene was sequenced. Primer sequences (from Folmer et al. 1994) were HCO2198: 5′-TAAACTTCAGGGTGACCAAAAAATCA-3′ and LCO1490: 5′-GGTCAACAAATCATAAAGATATTGG-3′.

Sequences were visually inspected, aligned and trimmed using Geneious v5.6.5 software (Drummond et al. 2012) with haplotypes exported as FASTA files. Arlequin v3 (Excoffier et al. 2005) and DnaSP v5 (Rozas and Rozas, 1999) were used to screen for duplicate haplotypes, determine haplotype frequencies, and quantify genetic diversity within populations (as nucleotide diversity and haplotype diversity). The hierarchical distribution of overall diversity was determined using an Analysis of Molecular Variation (AMOVA), as implemented in Arlequin. Genetic differentiation among populations was determined using (i) the average number of nucleotide substitutions per site or D_xy_ (MEGA v5; Tamura et al. 2011); and (ii) corrected pairwise difference between populations or P_xy_ (Arlequin). To determine the potential effect of isolation by distance, we performed a Mantel Test, as implemented in PopTools (Hood, 2010), to test for correlation between physical distance (Euclidian distance in km) and genetic distance (expressed as D_xy_ and P_xy_).

Additional COI sequences for *Ar. marginata, Ac. fulica* and *Ac. achatina* from West Africa (predominantly from Nigeria) were downloaded from GenBank for further phylogenetic analyses (Supplemental Table S2). A minimum spanning network was constructed for all *Ar. marginata, Ac. fulica* and *Ac. achatina* sequences using PopArt v1.7 (Leigh and Bryant 2015). Maximum likelihood (ML) and Bayesian inference (BI) phylogenetic analyses were conducted using Garli v2 (Zwickl 2006) and MrBayes v3.2 (Ronquist et al. 2012). The optimal nucleotide substitution model (TNr + I + Γ) was selected for these analyses by assessing the Akaike information criterion (AIC; Akaike 1974) in JMODELTEST v2.1 (Darriba et al. 2012). A consensus topology for the ML analysis was generated in PHYLIP v3.695 (Felsenstein 1989; Felsenstein 2009) following 1000 bootstrap replicates. The Bayesian analysis consisted of 10 million generations with a sampling frequency of 1000 and a burn-in of 0.25. It was assumed that the run reached convergence once the average standard deviation of split frequencies was below 0.01. A sequence from *Rumina decollata* was downloaded from Genbank (KC833730) for use as outgroup during the ML and BI analyses. All phylogenetic trees were viewed in FigTree v1.4 (Rambaut 2012).

A 406 bp fragment of the CO1 gene region was successfully amplified. For the Cameroonian specimens sequenced in this study, a total of 20 distinct haplotypes were identified for *Ar. marginata* and three haplotypes distinct to *Ac. fulica* (GenBank accession numbers KF512473-KF512495; Table 1). Within *Ar. marginata*, all populations showed a degree of haplotype diversity (Table 1), including very high levels of diversity evident from 4 distinct haplotypes in 4 snails from Ekondo, and 9 haplotypes in 11 snails from Mamfe. No diversity was however observed in 5 snails from the population from Buea (Table 1). For *Ac. fulica*, the population from the Tiko area showed moderate variation (3 haplotypes in 19 individuals) whereas the 20 Tombel individuals were fixed for a single haplotype. *Archachatina marginata* from Mamfe, Ekondo and Tombel displayed a haplotype diversity value of 0.95, 1.0 and 0.90, respectively (Table 1). A possible reason for the retained diversity in heavily harvested areas (Mamfe and Ekondo) might be the importation of snails from other localities to keep up with demand, and associated mixing of natural and introduced populations. However, there was no direct support for the notion of local mixing from the results of the phylogenetic analysis.

**Table 1.** Mitochondrial DNA haplotypes identified in the current study, GenBank accession numbers, haplotype frequencies and coefficients of genetic diversity for 7 Cameroonian populations of *Archachatina marginata* and two populations of *Achatina fulica*.

	*Ar. marginata*							*Ac. fulica*	
Haplotype	Buea	Ekona	Ekondo	Kumba	Mamfe	Muyuka	Tombel	Tiko	Tombel_Af
Hap01(KF512473)	1.0	0.500	–	0.500	–	–	0.400	–	–
Hap02(KF512474)	–	0.500	0.250	–	–	–	–	–	–
Hap03(KF512475)	–	–	0.250	–	–	–	–	–	–
Hap04(KF512476)	–	–	0.250	–	–	–	–	–	–
Hap05(KF512477)	–	–	0.250	–	–	–	–	–	–
Hap06(KF512478)	–	–	–	0.500	–	–	–	–	–
Hap07(KF512479)	–	–	–	–	0.273	–	–	–	–
Hap08(KF512480)	–	–	–	–	0.091	–	–	–	–
Hap09(KF512481)	–	–	–	–	0.091	–	–	–	–
Hap10(KF512482)	–	–	–	–	0.091	–	–	–	–
Hap11(KF512483)	–	–	–	–	0.091	–	–	–	–
Hap12(KF512484)	–	–	–	–	0.091	–	–	–	–
Hap13(KF512485)	–	–	–	–	0.091	–	–	–	–
Hap14(KF512486)	–	–	–	–	0.091	–	–	–	–
Hap15(KF512487)	–	–	–	–	0.091	–	–	–	–
Hap16(KF512488)	–	–	–	–	–	0.500	–	–	–
Hap17(KF512489)	–	–	–	–	–	0.500	–	–	–
Hap18(KF512490)	–	–	–	–	–	–	–	0.895	1.0
Hap19(KF512491)	–	–	–	–	–	–	–	0.053	–
Hap20(KF512492)	–	–	–	–	–	–	–	0.053	–
Hap21(KF512493)	–	–	–	–	–	–	0.200	–	–
Hap22(KF512494)	–	–	–	–	–	–	0.200	–	–
Hap23(KF512495)	–	–	–	–	–	–	0.200	–	–
Number of gene copies	5	2	4	2	11	2	5	19	20
Number of polymorphic loci	0	6	5	13	7	2	3	6	0
Polymorphic sites (n)	0	2	4	2	9	2	4	3	0
Nucleotide diversity (π)	0	0.015	0.008	0.032	0.005	0.005	0.003	0.001	0
Haplotype diversity (h)	0	1 ± 0.5	1 ± 0.177	1 ± 0.5	0.95 ± 0.066	1 ± 0.5	0.9 ± 0.161	0.21 ± 0.119	0

Results from AMOVA indicated that 79.35% of overall variation was found among populations of *Ar. marginata*, with 20.65% within populations. In *Ac. fulica*, however, only 0.28% of variation occurred between the two populations studied, with 99.72% of total variation found within populations. The D_xy_ values between conspecific population pairs were 0–0.039 for *Ar. marginata* and 0 for *Ac. fulica*. Differentiation expressed as P_xy_ showed a similar trend, with values of 0–0.160 among populations of *Ar. marginata* and 0 for the *Ac. fulica* populations. The comparative lack of diversity in *Ac. fulica* is most likely the signature of an invasion involving limited numbers.

Results from the Mantel test showed a positive correlation between both D_xy_ and P_xy_ and physical distance for *Ar. marginata*, with a correlation value of 0.481 in both cases. There was some indication of isolation by distance: the Mamfe population, which is most distant from the remaining populations (116–180 km), is also significantly different (from P_xy_ values) from all other populations. Similarly, population pairs Buea/Ekondo and Tombel/Ekondo, separated by 56–70 km, show evidence of significant differentiation (*F*_ST_ = 0.999; *p* = .003). By contrast, more closely clustered population pairs Buea/Ekona (12 km), Ekona/Muyuka (14 km), Tombel/Kumba (30 km), and Kumba/Ekona (52 km) do not show significant differentiation. No significant differentiation was observed for *Ac. fulica.* The observed *Ac. fulica* results correspond with that reported by Fontanilla et al. (2014). Historically, the model of gene-flow in edible snails from the forests of South West Cameroon was most likely based on a model of more-or-less continuous distribution, with isolation by distance only, following the model described by Wright (1943). However, the limited dispersal ability of the species would probably have resulted in the formation of genetic neighborhoods within the overall distribution area, with the size of such groups determined by dispersal ability. We note that Barker et al. (2013) detected genetic differentiation among populations of the South African terrestrial mollusk *Prestonella* over distances as short as 200–300 m.

The haplotype network (Figure 1) and both phylogenetic trees generated in the current study supports the occurrence of geographical genetic structuring for *Ar. marginata* and a homogeneity in *Ac. fulica* (Figure 2). The tree topologies are mostly similar, placing *Ar. marginata*, *Ac. fulica* and *Ac. achatina* in three distinct clades. The BI tree showed strong branch support for all major clades, with weaker support obtained from the ML tree analysis. Within the Cameroonian *Ar. marginata*, four lineages were identified: clade 1 consisting of Tombel, Buea, Ekona and Kumba haplotypes, clade 2 consisting of Ekona and Ekonda haplotypes, clade 3 consisting of Mamfe and Kumba haplotypes and clade 4 with the Mujuka haplotypes (Figure 2). All *Ac. fulica* haplotypes are grouped in one distinct clade. The Nigerian *Ar. marginata* sequences formed a clade sister to but separate from the Cameroonian specimens, with some sub-structuring observed as reported by Awodiran et al. (2015). The *Ar. marginata* and *Ac. achatina* specimens from Nigeria showed signs of shared haplotypes. We however believe that this could be either due to misidentification of the five relevant specimens during sampling by Awodiran et al. (2015) or presents a case of hybridization. Following the currently available data, we argue that the three Nigerian *Ar. marginata* specimens which grouped with the *Ac. achatina* clade is in fact misidentified *Ac. achatina* specimens. The same argument holds for the two Nigerian *A. achatina* specimens which strongly grouped with Nigerian *Ar. marginata* haplotypes.

**Figure 1.**
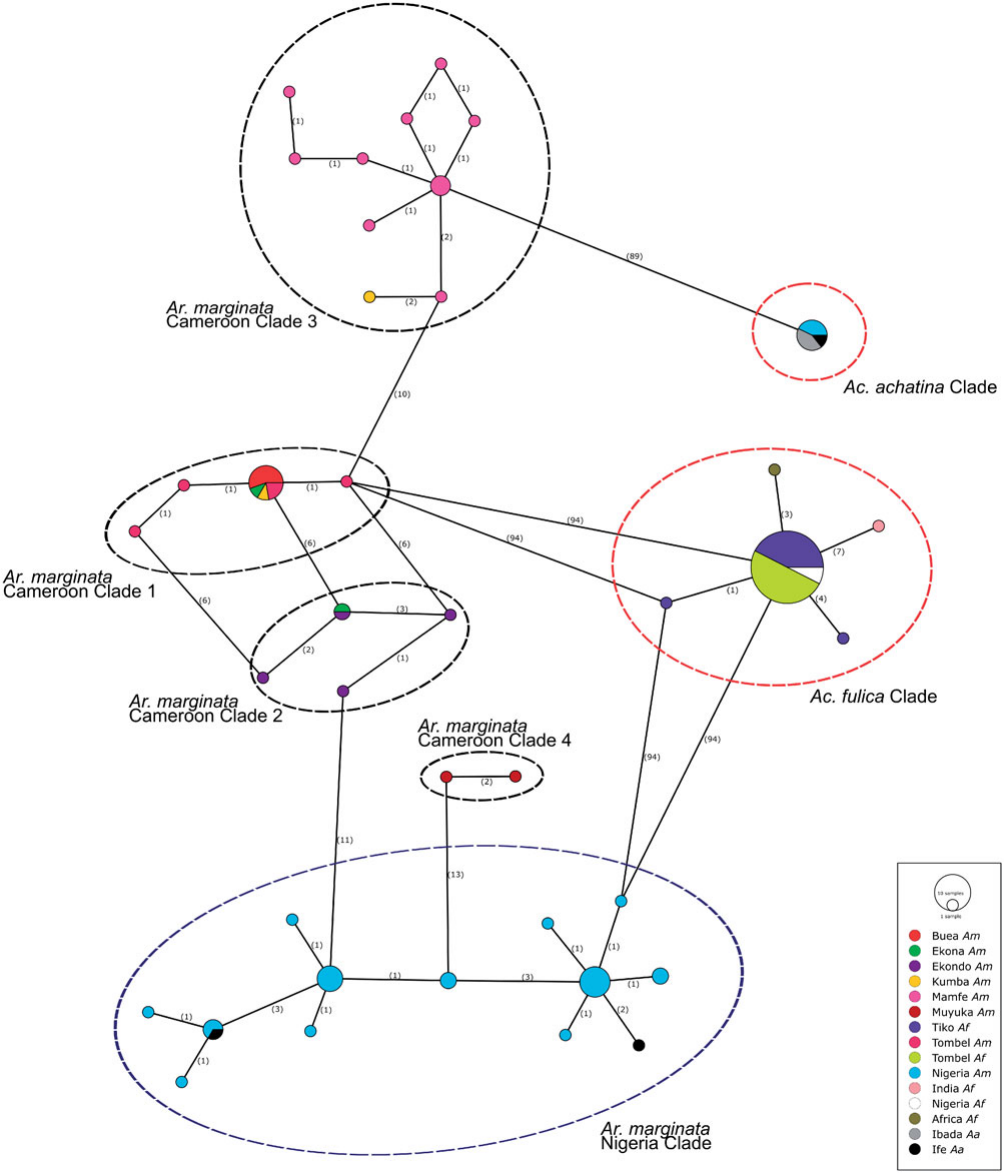
The minimum spanning network constructed for all *Archachatina marginata, Achatina fulica* and *Ac. achatina* sequences using PopArt. The Cameroonian *Ar. marginata* clades are identified by the black dashed ovals, the Nigerian *Ar. marginata* haplotypes are identified by the blue dashed ovals, with the *Ac. fulica* and *Ac. achatina* haplotypes in the red dashed ovals.

**Figure 2.**
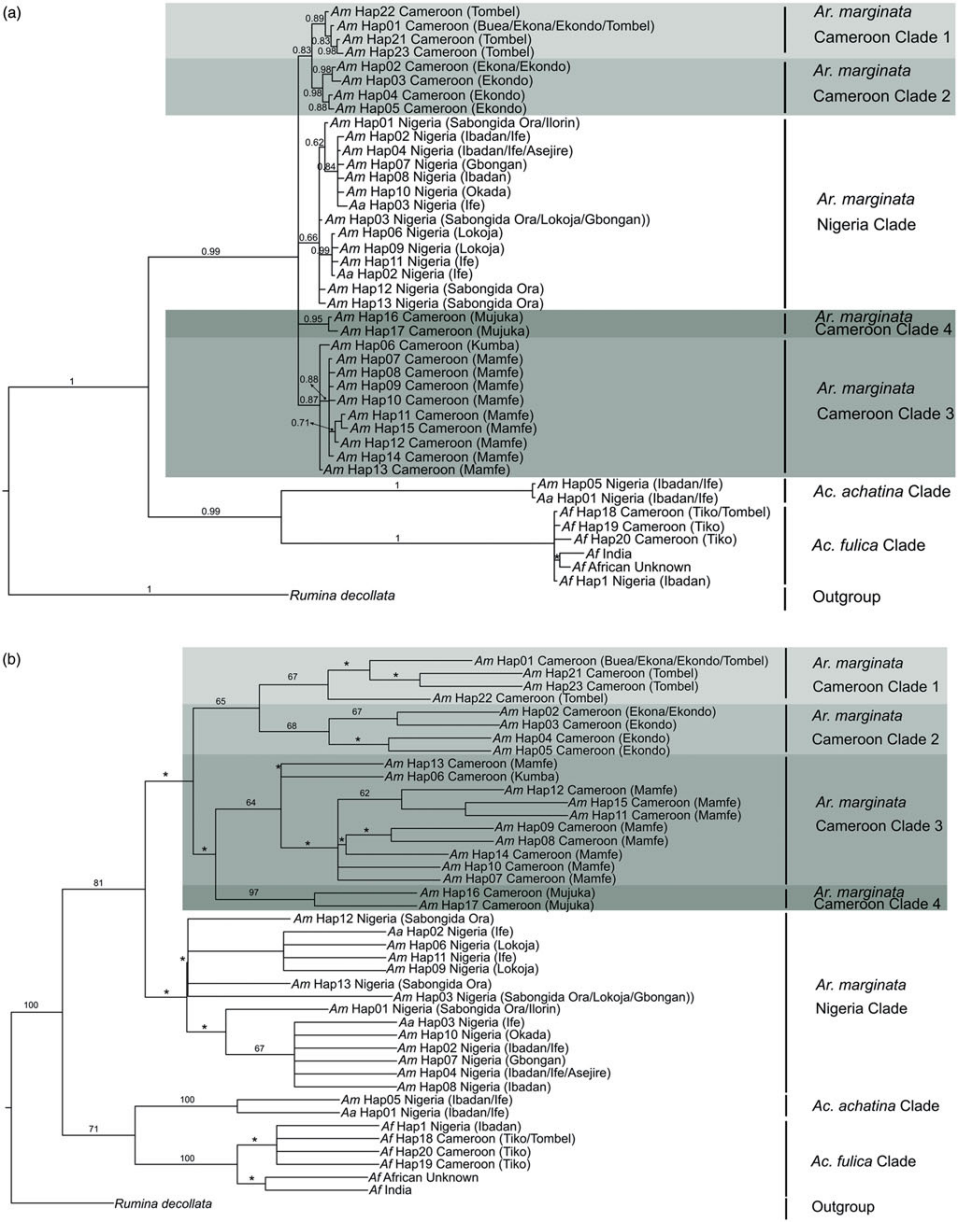
The (a) Bayesian inference (BI) tree and (b) maximum likelihood (ML) trees obtained for *Archachatina marginata*, *Achatina fulica* and *Ac. achatina* haplotypes from Cameroon and Nigeria. The Bayesian posterior probabilities below 0.6 and ML bootstrap support values below 60 are indicated by *. The observed lineages identified in the current study are shown to the right of each figure.

It is clear from this study that isolation by distance plays a significant role in determining the genetic structure of extant indigenous land snails in Cameroon, in line with the limited ability for dispersal in this species. The *Ar. marginata* population nevertheless display a diverse gene pool despite exploitation in recent years. In contrast, *Ac. fulica* shows much less diversity and structure, which is most likely the signature of an invasion involving low numbers.

## Supplementary Material

TMDN_A_1347837_Supplementary_Information.pdf
