# Supplementary material for: Current patterns of genetic diversity in indigenous and introduced species of land snails in Cameroon reflect isolation by distance, limited founder size and known evolutionary relationships
Source: Mitochondrial DNA B Resour. 2017 Jul 7;2(2):375–80. doi: 10.1080/23802359.2017.1347837 (PMC7800803; doi:10.1080/23802359.2017.1347837)
Supplement: TMDN_A_1347837_Supplementary_Information.pdf [file TMDN_A_1347837_SM7236.pdf]

Supplemental data for the manuscript:

Current patterns of genetic diversity in indigenous and introduced species of land snails in Cameroon reflect isolation by distance, limited founder size and known evolutionary relationships

Ivo N Woogeng [a, b, α], Willem G Coetzer [b, *], Kingsley A Etchu [c], Kenneth JN Ndamukong [a] and J Paul Grobler [b]

[a]Department of Zoology and Animal Physiology, University of Buea, Buea, Cameroon

[b]Department of Genetics, University of the Free State, Bloemfontein, South Africa

[c]Institute of Agricultural Research for Development (IRAD) Ekona, Cameroon

[*]**Corresponding author:** Dr Willem G. Coetzer

Department of Genetics,

University of the Free State,

PO Box 339,

Bloemfontein,

9300, South Africa

Email: coetzerwg@ufs.ac.za

Supplemental Tabel S1. Sample localities for the 31 *Archachatina marginata* and 39 *Achatina fulica* specimens collected for the current study.

Species	Number of samples	Devision	Sanpling locality	GPS coordinates
Achatina fulica	20	Kupe - Manengouba	Tombel	4°44'43.6"N 9°40'07.8"E
	19	Fako	Tiko	4°04'42.7"N 9°21'31.1"E
Archachatina marginata	11	Manyu	Mamfe	5°45'04.6"N 9°18'54.6"E
	2	Meme	Kumba	4°37'58.5"N 9°26'39.4"E
	5	Kupe - Manengouba	Tombel	4°44'43.6"N 9°40'07.8"E
	2	Fako	Muyuka	4°17'25.7"N 9°24'54.7"E
	2	Fako	Ekona	4°13'49.9"N 9°20'14.3"E
	5	Fako	Buea	4°08'51.7"N 9°14'48.9"E
	4	Ndian	Ekondo Titi	4°35'59.2"N 9°02'24.1"E

Supplemental Tabel S2. Additional *Ar. marginata*, *Achinatia fulica* and *Ac. achinatia* COI sequences downloaded from GenBank for use in subsequent phylogenetic analyses.

**Species**	**Country**	**Area/Town**	**Accession number**
Achatina achatina	Nigeria	Ibadan	KT290313
Achatina achatina	Nigeria	Ibadan	KT290314
Achatina achatina	Nigeria	Ibadan	KT290315
Achatina achatina	Nigeria	Ife	KT290311
Achatina achatina	Nigeria	Ife	KT290312
Achatina achatina	Nigeria	Ife	KT290316
Achatina fulica	Nigeria	Ibadan	KT290317
Achatina fulica	Nigeria	Ibadan	KT290318
Achatina fulica	Nigeria	Ibadan	KT290319
Achatina fulica	Africa	Unknown	AY148556
Achatina fulica	India	Unknown	KT583563
Archachatina marginata	Nigeria	Asejire	KT290287
Archachatina marginata	Nigeria	Gbongan	KT290283
Archachatina marginata	Nigeria	Gbongan	KT290286
Archachatina marginata	Nigeria	Gbongan	KT290288
Archachatina marginata	Nigeria	Gbongan	KT290303
Archachatina marginata	Nigeria	Ibadan	KT290296
Archachatina marginata	Nigeria	Ibadan	KT290299
Archachatina marginata	Nigeria	Ibadan	KT290300
Archachatina marginata	Nigeria	Ibadan	KT290306
Archachatina marginata	Nigeria	Ibadan	KT290309
Archachatina marginata	Nigeria	Ife	KT290291
Archachatina marginata	Nigeria	Ife	KT290292
Archachatina marginata	Nigeria	Ife	KT290297
Archachatina marginata	Nigeria	Ife	KT290298
Archachatina marginata	Nigeria	Ife	KT290301
Archachatina marginata	Nigeria	Ife	KT290302
Archachatina marginata	Nigeria	Ife	KT290305
Archachatina marginata	Nigeria	Ilorin	KT290307
Archachatina marginata	Nigeria	Lokoja	KT290285
Archachatina marginata	Nigeria	Lokoja	KT290293
Archachatina marginata	Nigeria	Lokoja	KT290295
Archachatina marginata	Nigeria	Lokoja	KT290304
Archachatina marginata	Nigeria	Okada	KT290294
Archachatina marginata	Nigeria	Sabongida Ora	KT290284
Archachatina marginata	Nigeria	Sabongida Ora	KT290289
Archachatina marginata	Nigeria	Sabongida Ora	KT290290
Archachatina marginata	Nigeria	Sabongida Ora	KT290308
Archachatina marginata	Nigeria	Sabongida Ora	KT290310
